# Insights into the genes involved in the ethylene biosynthesis pathway in *Arabidopsis thaliana* and *Oryza sativa*

**DOI:** 10.1186/s43141-020-00083-1

**Published:** 2020-10-19

**Authors:** Mostafa Ahmadizadeh, Jen-Tsung Chen, Soosan Hasanzadeh, Sunny Ahmar, Parviz Heidari

**Affiliations:** 1 Minab Higher Education Center, University of Hormozgan, Bandar Abbas, Iran; 2 Department of Life Sciences, National University of Kaohsiung, Kaohsiung, 811 Taiwan; 3 Department of Horticultural Sciences, Faculty of Agriculture, Shahrood University of Technology, Shahrood, Iran; 4 National Key Laboratory of Crop Genetic Improvement, College of Plant Science and Technology, Huazhong Agricultural University, Wuhan, 430070 Hubei China; 5 Department of Agronomy and Plant Breeding, Faculty of Agriculture, Shahrood University of Technology, Shahrood, Iran

**Keywords:** Cis-acting elements, miRNAs, Post-transcriptions modifications, Ligand binding site, Pathway study

## Abstract

**Background:**

Ethylene is a gaseous plant hormone that acts as a requisite role in many aspects of the plant life cycle, and it is also a regulator of plant responses to abiotic and biotic stresses. In this study, we attempt to provide comprehensive information through analyses of existing data using bioinformatics tools to compare the identified ethylene biosynthesis genes between Arabidopsis (as dicotyledonous) and rice (as monocotyledonous).

**Results:**

The results exposed that the Arabidopsis proteins of the ethylene biosynthesis pathway had more potential glycosylation sites than rice, and 1-aminocyclopropane-1-carboxylate oxidase proteins were less phosphorylated than 1-aminocyclopropane-1-carboxylate synthase and S-adenosylmethionine proteins. According to the gene expression patterns, S-adenosylmethionine genes were more involved in the rice-ripening stage while in Arabidopsis, *ACS2*, and 1-aminocyclopropane-1-carboxylate oxidase genes were contributed to seed maturity. Furthermore, the result of miRNA targeting the transcript sequences showed that ath-miR843 and osa-miR1858 play a key role to regulate the post-transcription modification of S-adenosylmethionine genes in Arabidopsis and rice, respectively. The discovered cis- motifs in the promoter site of all the ethylene biosynthesis genes of *A. thaliana* genes were engaged to light-induced response in the cotyledon and root genes, sulfur-responsive element, dehydration, cell cycle phase-independent activation, and salicylic acid. The ACS4 protein prediction demonstrated strong protein-protein interaction in Arabidopsis, as well as, *SAM2*, *Os04T0578000*, *Os01T0192900*, and *Os03T0727600* predicted strong protein-protein interactions in rice.

**Conclusion:**

In the current study, the complex between miRNAs with transcript sequences of ethylene biosynthesis genes in *A. thaliana* and *O. sativa* were identified, which could be helpful to understand the gene expression regulation after the transcription process. The binding sites of common transcription factors such as MYB, WRKY, and ABRE that control target genes in abiotic and biotic stresses were generally distributed in promoter sites of ethylene biosynthesis genes of *A. thaliana*. This was the first time to wide explore the ethylene biosynthesis pathway using bioinformatics tools that markedly showed the capability of the in silico study to integrate existing data and knowledge and furnish novel insights into the understanding of underlying ethylene biosynthesis pathway genes that will be helpful for more dissection.

## Background

The gas ethylene has been known as a signaling molecule, which regulates stress responses and various developmental processes in plants [1, 2], such as a boost of fruit ripening, petal and leaf abscission, flower senescence, incitement of root initiation, and prevention of seedling elongation [3]. Besides, ethylene is produced in response to environmental stresses [3], consisting of wounding [4], flooding [5], bacteria, viruses, fungi, nematodes, and insects [6]. Ethylene has been known as a regulated hormone under stress conditions [7], and several studied ecotypes on stress-responsive genes revealed various basal expression levels [8]. Increment the production of ethylene works as a signaling mechanism with intense physiological outcomes [1, 9, 10]. Ethylene is synthesized from methionine through its transformation to S-adenosylmethionine that it is converted via the enzyme 1-aminocyclopropane-1-carboxylate synthase into methylthioadenosine and 1-aminocyclopropane-1-carboxylic acid (ACC) as the precursor of ethylene [11]. 1-aminocyclopropane-1-carboxylic acid (ACC) is oxidized to HCN, CO_2_, and C_2_H_4_ by ACC oxidase (ACO) [12]. Besides, ACC could be turned from transformation to ethylene by forming the conjugate N-malonyl-ACC [13]. Bleecker et al [11] the 1-aminocyclopropane-1-carboxylate synthase (ACS) activity is regulated at the transcriptional and post-transcriptional levels [1, 9]. Owing to the influence of ethylene on senescence and ripening, vast vegetables, fruit, and flowers are lost. Therefore, as a reversible manner, the endeavor has been done to delay or prevent fruit ripening. The activity of ACC synthase has been illustrated with antisense RNA experiments in the role of the rate-limiting phase in ethylene synthesis [14].

The natural diversity of ethylene production suggests that plants by fine-tuning biosynthetic of ethylene and signaling pathways can adapt to different environments. Observation of some stress-responsive genes revealed that this adaptation could be associated to modify the expression of ACS genes via epigenetic modifications [8, 15]. Moreover, it has been demonstrated that ethylene influence the transcription and translation of many genes which are related to ripening [16], in tomato, at least eight ACS genes have been recognized [17]. The Arabidopsis genome consists of 12 putative ACS-like genes, further, from the ACS genes, *ACS3* was identified as pseudogene by a short sequence, besides, *ACS12* and *ACS10* encode an aminotransferase sans the catalytic activity of ACS [18]. The nine remaining ACS genes encode an ACS proteins group which could be categorized into 3 types, according to the absence or presence of putative phosphorylation sites at the proteins C-terminal extension [19, 20]. Type-1 ACS proteins consist of an almost lengthy C-terminal domain that shares the target sites and extremely conserved sequences for a calcium-dependent protein kinase (CDPK) and mitogen-activated protein kinase (MAPK) [21–24], while, type-2 have only the anticipated CDPK phosphorylation site. Nonetheless, type-2 ACS proteins consist of an exclusive regulatory motif named a target of ethylene overproducer (ETO1) (TOE) that overlaps by the CDPK target site. Besides, TOE motif mediates interaction by ETO1 E3 ligase, and its two paralogs, ETO1-Like (EOL1 and EOL2), also it is needed for type-2 ACS degradation [24–28]; type-3 ACS contains only a short expansion of amino acids in the C-terminal domain, and no target sites for a MAPK and CDPK [19, 24]**.** Another plant that was selected for this study was rice as monocotyledonous, rice is the main staple cereal that feeds almost half of the world’s population. Owing to the enhancing worldwide demand of the growing population, approximately, 50% enhance in production of rice will be needed [10]. Rice has the shortest genome of the main cereals and wealthy genetic diversity. Moreover, the sequence of rice whole-genome furnishes the basis to identify the homologous genes for other crops [29, 30]. Its sustainability and productivity are crucially threatened via several biotic and abiotic stresses such as submergence, drought, salinity, and chilling, but ethylene plays an initial role in adopting plants under stress conditions [20, 31]. In deep water rice, it has been demonstrated that *OsACO1* is involved in the internode elongation, also, submergence enhances the ACO enzyme activity and levels of *OsACO1* mRNA [32, 33]. The expression of *OsACO3* and *OsACO2* genes in etiolated rice seedlings was also revealed to be diversely controlled via auxin and ethylene [34].

Ethylene is the main hormone, which controls many physiological pathways. Ethylene has been suggested to be more potent versus necrotrophic pathogens (like *B. cinerea*) than against biotrophic pathogens. Ethylene insensitive mutants etr1, ein3, and ein2, display increased susceptibility to *B. cinerea* [35, 36]. Also, plants that overexpress transcription factors associated with the jasmonic acid and ethylene pathways expose an enhanced resistance to different necrotrophs [37–39]. In Arabidopsis, overexpression of *AP*_*2*_*C*_*1*_ that encodes a Thr or Ser protein type 2C phosphatase decreased production of ethylene and compromises resistance to the necrotrophic pathogen *B. cinerea* [40]. On the other hand, the ACSs could be adjusted via putative endogenous signal receptors like phytohormones and intracellular accumulation of secondary metabolites, such as calcium [3]. Moreover, usage of ACC or ethylene could enhance plant salinity tolerance, mainly by increasing the expression of reactive oxygen species scavengers [41–43]. The expression of ACO genes from various species is also associated with the ethylene biosynthesis rate, as well as ACS, and the transcript levels of multiple ACO genes are regulated under stress conditions [44, 45]. There are some ethylene response factors (ERFs) gene family, across the environmental stress-responsive genes, the mRNA levels of various ERF are controlled via several molecules produced and hormones in various stress conditions [46]. Ethylene plays a biphasic role, inhibiting and stimulating growth dependent upon the species, developmental stages of organs or tissue, and environmental conditions [47, 48]. Ethylene prevents hypocotyl elongation by the switch on the transcription factors ethylene response factor 1 (ERF1) [49–51] and waved-dampened 5 (WDL5) in Arabidopsis [52] in the low light severity or dark. Transcription factor hypocotyl 5 (HY5) also gets involved in this action that is degraded via the E3 ligase constitutive photomorphogenic 1 (COP1) [53]. Adjustment of the ACS transcript levels seems to be a critical mechanism to control the alteration of plant ethylene production. Nonetheless, recent studies put forward that posttranslational modifications, like ubiquitination and phosphorylation, provide as a momentous mechanism to adjust the stability of the ACS proteins that will be led to regulate the levels of ethylene in plants [24, 54, 55].

Considering the riches of the genome sequence information of rice and Arabidopsis which is supplying a valuable resource to study and dissect ethylene biosynthesis genes in monocotyledons (rice) and dicotyledons (Arabidopsis). The genes that are responsible for the biosynthesis of ethylene in Arabidopsis and rice were retrieved delicately. Regarding the importance of the post transcription and translation modifications, the study of the phosphorylation, glycosylation, and miRNA target ethylene biosynthesis genes will be useful. Besides, the cis-regulatory elements in promoter regions of ethylene biosynthesis genes will give a better understanding of the regulation of these genes expression. Moreover, the perception of cis-acting regulatory elements can help to change gene expression patterns through plant genetic engineering approaches to avoid biotic and abiotic stress damages. The present study was the first study to provide comprehensive information and a wide analysis of ethylene biosynthesis genes by available bioinformatics tools for dissection of promoter regions, mRNA, and protein sequences of ethylene biosynthesis genes of two important model plants including Arabidopsis and rice.

## Methods

### Retrieve the ethylene genes and sequence analysis

The involved genes identification for the pathway of ethylene biosynthesis in Arabidopsis and rice were performed using the Plantcyc (https://www.plantcyc.org/). The sequences of transcript and polypeptide of all involving genes in ethylene biosynthesis of *Arabidopsis thaliana* from the Arabidopsis Information Resource (TAIR) (https://www.arabidopsis.org/) and *Oryza sativa* from Rice Genome Annotation Project Database (http://rice.plantbiology.msu.edu/index.shtml) were retrieved, respectively [56].

### Biochemical characteristics

Prediction of biochemical traits such as molecular weight (MW), isoelectric point (pI), aliphatic index, instability index, and grand average of hydropathy (GRAVY) was done by polypeptide sequences of ethylene biosynthesis genes and ProtParam tool of Expasy database [57] (https://web.expasy.org/protparam/). The subcellular location of proteins was predicted using Plant-mPLoc (https://www.csbio.sjtu.edu.cn/bioinf/plant-multi) for both *Arabidopsis thaliana* and *Oryza sativa*.

### Evolutionary analysis

The full length of the amino acid sequence of all predicted SAM, ACS, and ACO proteins of rice and Arabidopsis were used to align using ClustalX. The phylogenetic tree was constructed using the neighbor-joining method of clustal omega (https://www.ebi.ac.uk/Tools/msa/clustalo/).

### 3D protein structure prediction and domain analysis

Three-dimensional (3D) protein structure and ligand-binding site of SAM, ACS, and ACO genes were predicted using the homology modeling of SWISS-MODEL [58]. Also, protein sequences of studied genes were analyzed using the MOTIF Search program https://www.genome.jp/tools/motif/ for finding the conserved motifs and domains.

### Gene expression analysis and identification of miRNA targets

Microarray expression of intended genes in *Arabidopsis thaliana* and *Oryza sativa* under biotic and abiotic stresses and hormones treatment were obtained from the Genevistigator database [59]. The Affymetrix rice genome array (2836 samples) and Affymetrix Arabidopsis ATH1 genome array (10615 samples) were selected to study the expression patterns of ethylene biosynthesis genes in rice and Arabidopsis, respectively. The psRNATarget server (http://plantgrn.noble.org/psRNATarget/) applied to find existing miRNAs of *Arabidopsis thaliana* and *Oryza sativa* at 3.5 expectation level by searching all the transcript sequences of desired genes in Arabidopsis and rice [60].

### Prediction of putative Cis-elements

To identify the probable cis-regulatory elements, the promoter sequences (1500 bp upstream of transcription start site) of ethylene biosynthesis pathway genes in Arabidopsis and rice were perused by plantpan2 database (http://plantpan2.itps.ncku.edu.tw/index.html) [61].

### Prediction the glycosylation and phosphorylation sites

The NetNGlyc 1.0 server (http://www.cbs.dtu.dk/services/NetNGlyc/) was utilized to determine potential N-glycosylation sites [62]. The predictable phosphorylation sites were identified by NetPhos 3.1 server (http://www.cbs.dtu.dk/services/NetPhos/) [63].

## Results

### Biochemical characteristics SAM, ACS, and ACO genes in *Arabidopsis thaliana* and *Oryza sativa*

**Fig. 1:**
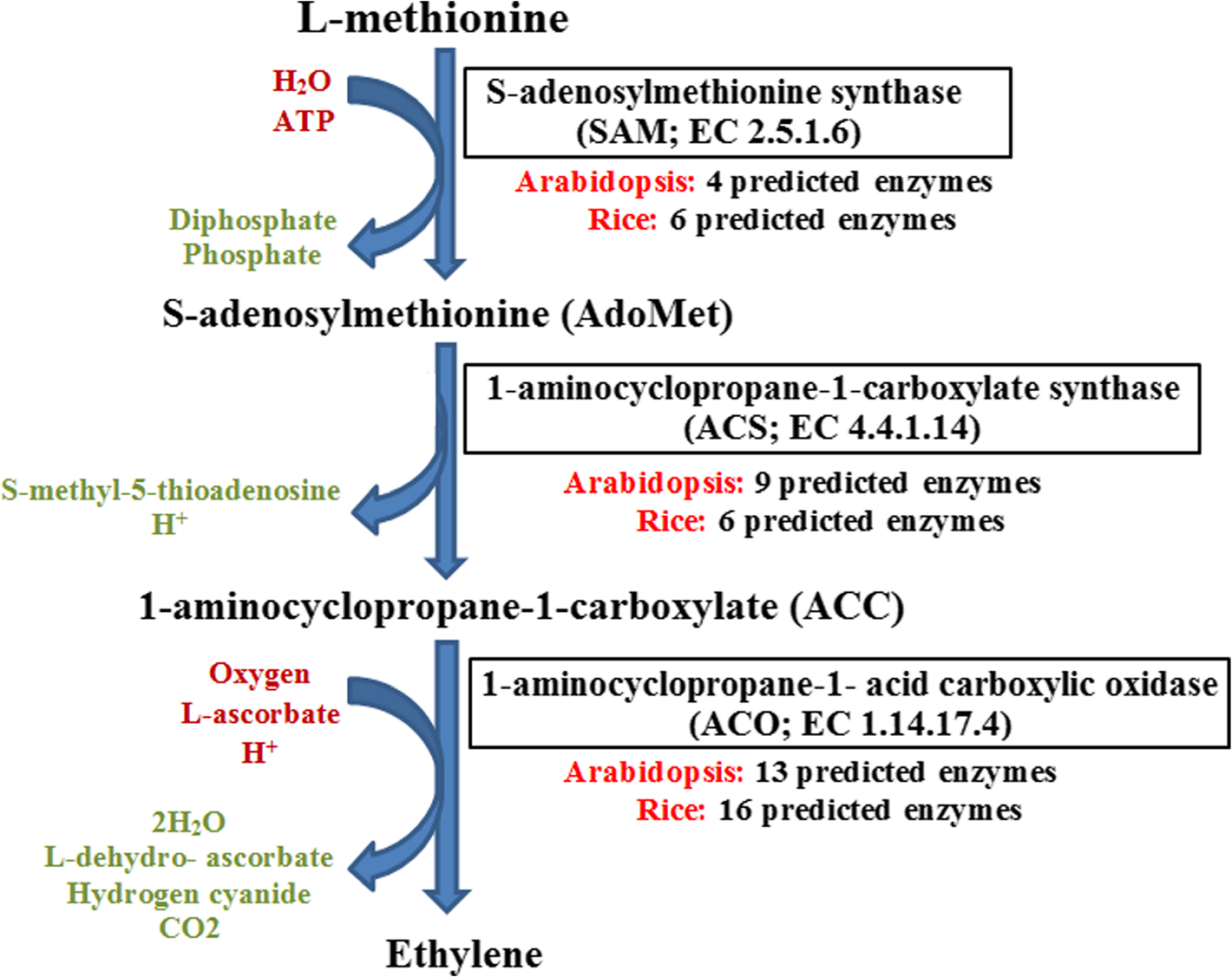
Ethylene biosynthesis pathway in plant [11, 13]

The genes which are involved in the pathway of biosynthesis of ethylene in Arabidopsis and rice were detected by the Plantcyc database. According to the ethylene biosynthesis pathway, 26 and 28 engaging enzymes were predicted in *A. thaliana* and *O. sativa*, respectively (Fig. 1). Besides, from 26 identified genes 4, 9, and 13 were identified as methionine adenosyltransferase (SAM), aminocyclopropane-1-carboxylate synthase (ACS), and aminocyclopropane-1-carboxylate oxidase (ACO), respectively in *A. thaliana*, as well as, number of 6, 6, and 16 predicted genes were involved in SAM, ACS, and ACO, respectively, in *O. sativa*. The number of ACO engaged genes was more than the genes which were involved in SAM and ACS (Fig. 1, Table 1).

**Table 1:** The properties of ethylene biosynthesis genes in *Arabidopsis thaliana* and *Oryza sativa*

Locus ID	Gene name	Length (aa)	MW (kDa)	pl	Instability index	Aliphatic index	GRAVY	Subcellular localization
*AT2G36880*	SAM3	390	42.50	5.76	Stable	86.26	−0.209	Chloroplast
*AT4G01850*	SAM2	393	43.26	5.67	Stable	83.74	−0.236	Chloroplast
*AT3G17390*	SAM4	393	42.80	5.51	Stable	79.34	−0.353	Chloroplast
*AT1G02500*	SAM1	393	43.16	5.51	Stable	85.04	−0.255	Chloroplast
*LOC_Os01g22010*	SAM	394	42.90	5.68	Stable	81.14	−0.264	Chloroplast
*LOC_Os01g18860*	SAM	396	43.31	5.22	Stable	82.7	−0.274	Chloroplast
*LOC_Os05g04510*	SAM	396	43.22	5.74	Stable	82.45	−0.289	Chloroplast
*LOC_Os02g57990*	SAM	317	34.27	5.98	Stable	96.53	0.036	Golgi apparatus
*LOC_Os07g29440*	SAM	164	17.80	4.77	Unstable	75.49	−0.268	Cytoplasm
*LOC_Os01g10940*	SAM	164	17.85	4.78	Stable	75.49	−0.291	Cytoplasm
*AT3G61510*	ACS1	488	55.00	7.18	Unstable	80.33	−0.306	Chloroplast
*AT1G01480*	ACS2	496	55.53	7.2	Stable	83.52	−0.205	Chloroplast
*AT2G22810*	ACS4	474	53.80	8.5	Stable	87.46	−0.201	Cytoplasm
*AT5G65800*	ACS5	470	53.31	7.55	Unstable	83.71	−0.351	Cytoplasm
*AT4G37770*	ACS8	496	53.37	8	Stable	81.72	−0.339	Cytoplasm
*AT4G26200*	ACS7	447	50.67	5.94	Unstable	82.75	−0.386	Chloroplast
*AT4G11280*	ACS6	495	55.52	6.23	Unstable	83.31	−0.337	Chloroplast
*AT4G08040*	ACS11	460	51.80	6.34	Stable	80.77	−0.271	Cytoplasm
*AT3G49700*	ACS9	470	53.17	6.73	Stable	80.28	−0.36	Cytoplasm
*LOC_Os05g10780*	ACS	437	47.73	6.1	Unstable	84.92	−0.111	Chloroplast
*LOC_Os01g09700*	ACS	510	55.44	7.16	Unstable	85.98	−0.077	Chloroplast
*LOC_Os05g25490*	ACS	496	53.51	5.66	Stable	85.65	−0.077	Chloroplast
*LOC_Os06g03990*	ACS	542	59.48	8.99	Unstable	87.51	−0.148	Chloroplast
*LOC_Os03g51740*	ACS	487	53.14	8.49	Unstable	85.17	−0.115	Cytoplasm
*LOC_Os04g48850*	ACS	483	54.34	6.83	Unstable	80.6	−0.259	Chloroplast
*AT3G46500*	ACO	251	28.48	6.38	Stable	85.66	−0.31	Cytoplasm
*AT3G49620*	ACO	357	40.70	6.22	Stable	80	−0.366	Cytoplasm
*AT1G35190*	ACO	329	37.64	5.47	Stable	89.75	−0.226	Cytoplasm
*AT3G49630*	ACO	332	37.46	5.65	Stable	79.12	−0.409	Cytoplasm
*AT4G16765*	ACO	247	27.75	5.33	Stable	89.22	−0.219	Cytoplasm
*AT3G50210*	ACO	332	37.22	5.16	Stable	89.15	−0.152	Cytoplasm
*AT4G16770*	ACO	258	29.08	5.66	Unstable	84.25	−0.266	Cytoplasm
*AT3G46490*	ACO	330	37.61	5.75	Stable	78.22	−0.381	Cytoplasm
*AT1G77330*	ACO5	307	34.95	5.05	Stable	77.42	−0.441	Cytoplasm
*AT2G19590*	ACO1	310	35.20	6.17	Stable	76.84	−0.574	Cytoplasm
*AT1G62380*	ACO2	320	36.18	4.98	Stable	74.55	−0.487	Cytoplasm
*AT1G12010*	ACO	320	36.53	5.09	Stable	78.56	−0.498	Cytoplasm
*AT1G05010*	ACO4	323	36.68	5.24	Stable	81.97	−0.43	Cytoplasm
*LOC_Os10g37899*	ACO	544	59.36	6.66	Unstable	79.15	−0.363	Chloroplast, Cytoplasm
*LOC_Os04g55070*	ACO	326	35.80	5.34	Unstable	83.22	−0.247	Cytoplasm
*LOC_Os05g35000*	ACO	222	24.68	11.18	Unstable	70.45	−0.662	Chloroplast
*LOC_Os09g07450*	ACO	202	22.93	5.45	Stable	93.66	−0.311	Cytoplasm
*LOC_Os08g33020*	ACO	286	31.16	11	Unstable	73.11	−0.698	Chloroplast, Nucleus
*LOC_Os01g61440*	ACO	394	40.50	4.96	Unstable	80.61	−0.09	Cytoplasm
*LOC_Os09g27820*	ACO	322	36.44	4.99	Unstable	82.7	−0.352	Cytoplasm
*LOC_Os02g53180*	ACO	344	38.29	6.81	Unstable	78.63	−0.368	Cytoplasm
*LOC_Os05g05680*	ACO	308	34.58	5.11	Stable	71.59	−0.452	Cytoplasm
*LOC_Os06g37590*	ACO	293	33.62	5.88	Unstable	80.82	−0.423	Cytoplasm
*LOC_Os01g39860*	ACO	312	34.03	5.21	Stable	81.7	−0.206	Cytoplasm
*LOC_Os09g27750*	ACO	322	36.34	5.2	Unstable	84.25	−0.315	Cytoplasm
*LOC_Os10g37880*	ACO	308	34.24	5.35	Stable	83.64	−0.25	Cytoplasm
*LOC_Os11g08380*	ACO	309	34.75	4.93	Unstable	79.61	−0.403	Cytoplasm
*LOC_Os09g07020*	ACO	435	48.94	7.1	Stable	77.49	−0.315	Cytoplasm
*LOC_Os05g05670*	ACO	157	17.53	5.07	Stable	73.95	−0.522	Cytoplasm

The total number of amino acids in studied genes ranged from 251–490 aa that *AT3G46500* was the smallest protein involved in ACO and *AT4G37770* was the largest predicted protein at ACS in Arabidopsis (Table 1). Also, the length of amino acids varied from 157 to 544 aa in rice that *LOC_Os05g05670* was the smallest protein, and *LOC_Os10g37899* was the largest protein both engaged with ACO. Furthermore, the high length of proteins was contributed to the ACS in both Arabidopsis and rice (Table 1). The GRAVY values of *A. thaliana* were varied between −0.152 (*AT3G50210*) a − 0574 (*AT2G19590*), besides, the GRAVY range was from −0.077 to 0.036 in *O. sativa* (Table 1).

Molecular weight (MW) of proteins in *A. thaliana* varied between 27.75 and 55.53 kDa while in *O. sativa*, they ranged between 17.53 and 59.48 kDa. Isoelectric points (pI) of proteins in *A. thaliana* ranged from 5.16 (*AT3G50210*) to 8.5 (*AT2G22810*) while in *O. sativa*, they varied from 4.77 (*LOC_Os07g29440*) to 11.18 (*LOC_Os05g35000*). Most of the predicted proteins in *A. thaliana* were stable except the proteins involved in ACS; however, in rice, the larger part of the proteins that contribute to the ACS and ACO was unstable (Table 1). The range of the aliphatic index was from 74.55 (*AT1G62380*) to 89.75 (*AT1G35190*) in *A. thaliana*, further, the aliphatic index was varied in *O. sativa* between 70.45 (*LOC_Os05g35000*) and 96.53 (*LOC_Os02g57990*). The lowest and highest aliphatic indices presented in ACO and SAM rice predicted proteins, respectively (Table 1). The predicted localization of the proteins was diverse and included the chloroplast, Golgi apparatus, cytoplasm, and nucleus (Table 1). The majority of SAM proteins were localized to the chloroplast in both *A. thaliana* and *O. sativa* except *LOC_Os02g57990* (Golgi apparatus), *LOC_Os07g29440*, and *LOC_Os01g10940* (cytoplasm) in *O. sativa* (Table 1). The ACS predicted proteins were localized in the chloroplast and cytoplasm. Besides, most of the ACO proteins were associated with the cytoplasm in both *A. thaliana* and *O. sativa*; however, the *LOC_Os10g37899* and *LOC_Os08g33020* were located in the chloroplast as well as cytoplasm and nucleus, respectively (Table 1). The results revealed that genes involved in ethylene biosynthesis from rice are more varied than these genes from Arabidopsis.

### Phylogenetic relationship

**Fig. 2:**
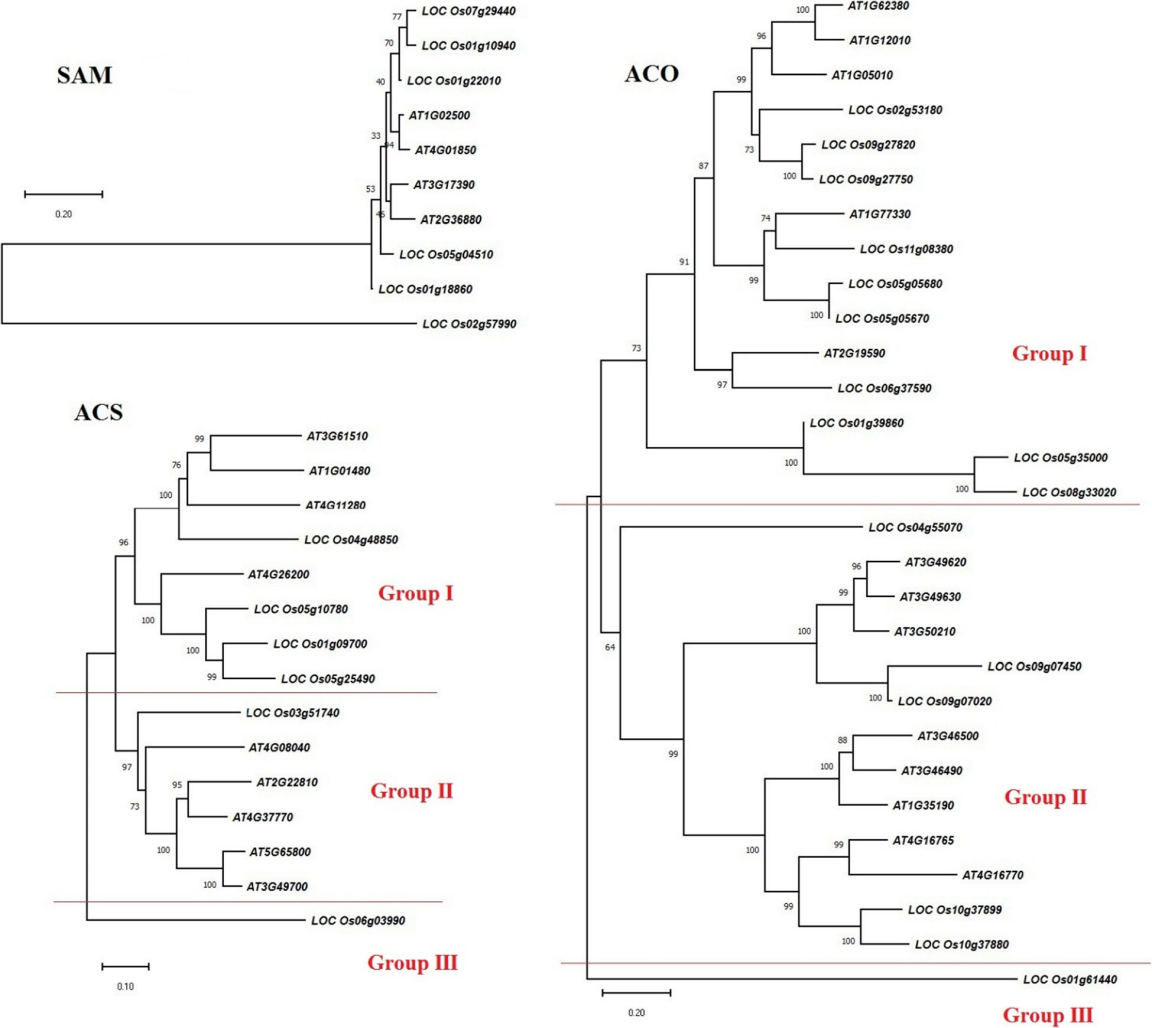
Phylogenetic analysis of methionine adenosyltransferase (SAM), aminocyclopropane-1-carboxylate synthase (ACS), and aminocyclopropane-1-carboxylate oxidase (ACO) enzymes of Arabidopsis, tomato and rice based on amino acid sequences using the neighbor-joining method of clustal omega (https://www.ebi.ac.uk/Tools/msa/clustalo/)

To investigate the evolutionary relationships among involved genes of the ethylene biosynthesis pathway, we constructed the phylogenetic tree by the rooted neighbor-joining method using the amino acid sequences from Arabidopsis, and rice (Fig. 2). According to the phylogenic tree, the *LOC_Os2g57990* that was predicted to have Golgi apparatus localization had more genetic distance than other rice-SAM genes. Also, two rice-SAM proteins (*LOC_Os07g29440* and *LOC_Os01g10940*) which were predicted to cytoplasm localization had high similarity based on amino acid sequences (Table 1, Fig. 2). According to the evolutionary relationships among SAM proteins, it seems that rice SAM genes had more variation than Arabidopsis SAM genes (Fig. 2). ACS proteins were clustered into three groups that 8 of 15 ACSs were located in the first group. Interestingly, a predicted ACS protein of rice (*LOC_Os06g03990*) had a high distance with others (Fig. 2). In the first group, *AT3G61510*, *AT1G01480, AT4G11280* (ACS6), and *LOC_Os04g8850* had more distance than other ACS proteins from rice and Arabidopsis and it was worth noting that these proteins were predicted to locate in the chloroplast (Table 1, Fig. 2). Also, the evolutionary relationships of ACO proteins revealed that they could be clustered into three groups based on the similarity of amino acid sequences. In this way, the first group contained 15 ACOs while 13 ACOs were clustered into second. The *LOC_Os01g61440* had more genetic distance than other the studied ACOs. All Arabidopsis ACO proteins were predicted to have cytoplasm localization, but rice ACO proteins were different in terms of protein localization. Also, phylogenetic analysis between ACO proteins showed that rice ACO proteins had high variation than Arabidopsis ACO proteins (Table 1, Fig. 2).

### Protein structure and domain analysis

**Fig. 3:**
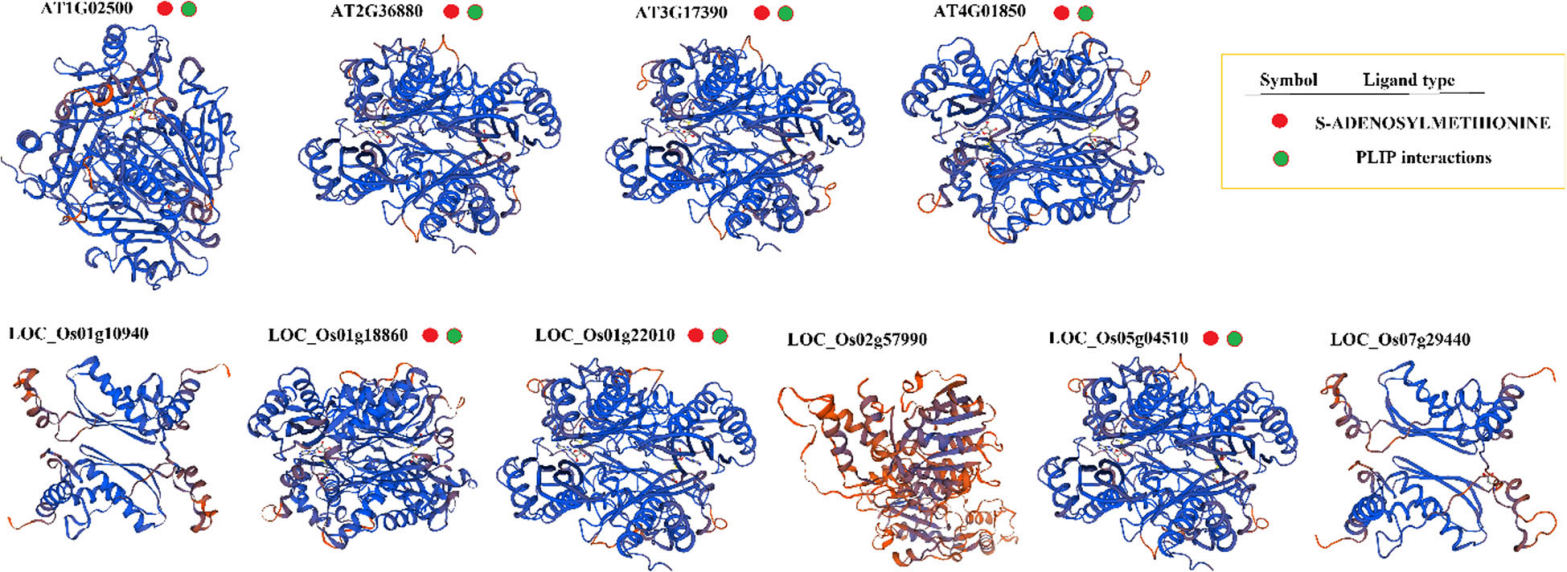
The predicted 3D model structure and ligand type of methionine adenosyltransferase (SAM) proteins in Arabidopsis and rice using SWISS-MODEL [58]

**Fig. 4:**
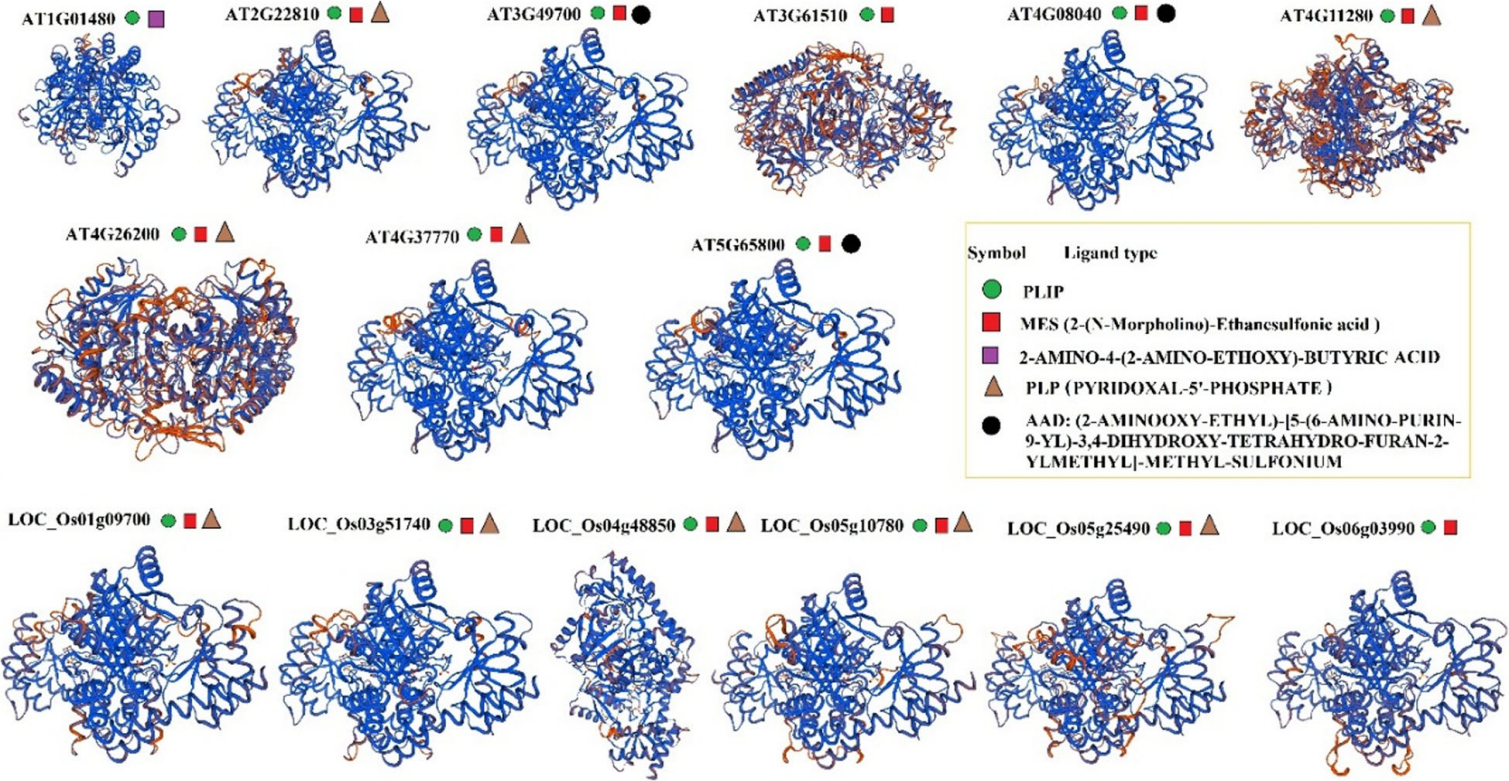
The predicted 3D model structure and ligand type of aminocyclopropane-1-carboxylate synthase (ACS) proteins in Arabidopsis and rice using SWISS-MODEL [58]

**Fig. 5:**
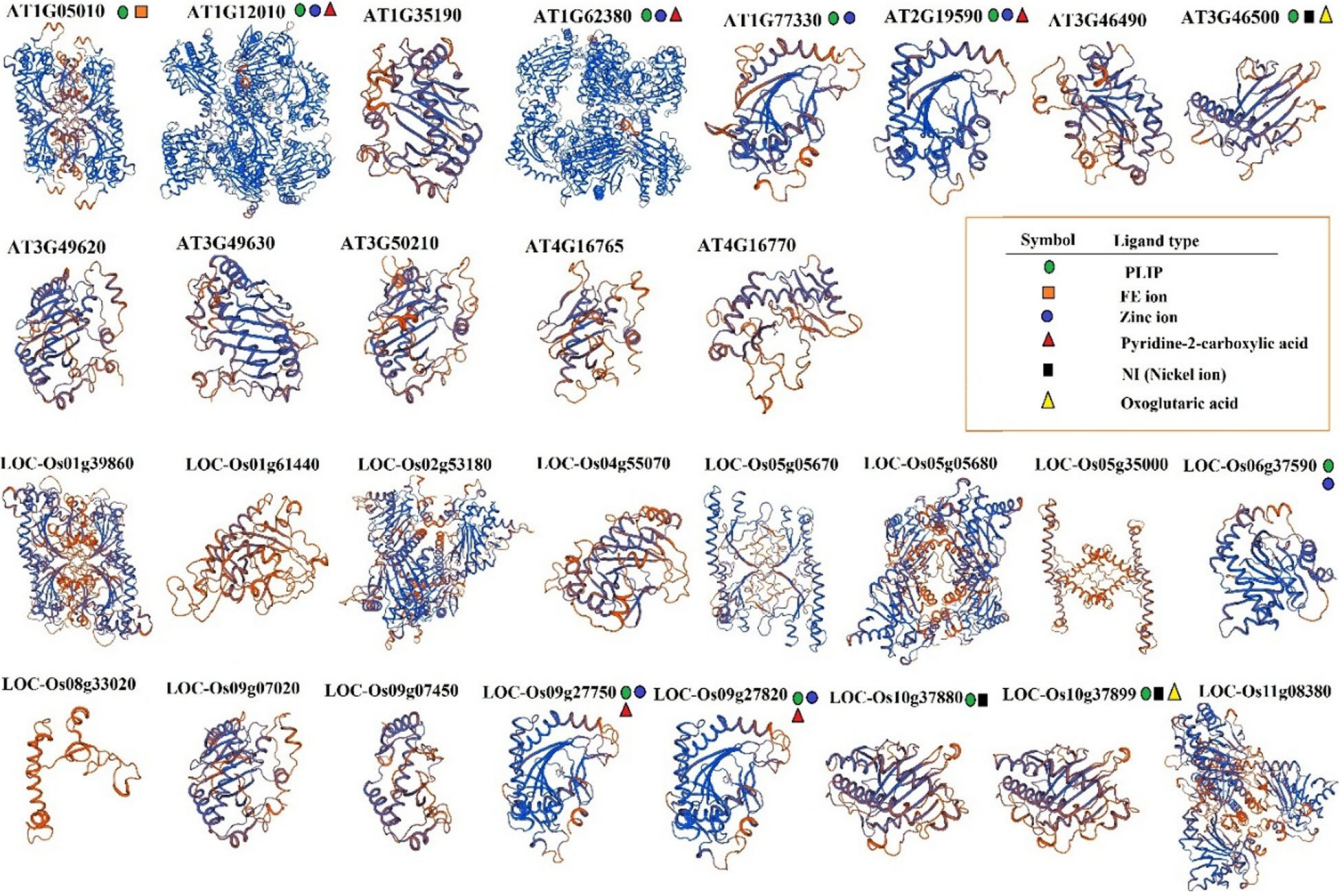
The predicted 3D model structure and ligand type of aminocyclopropane-1-carboxylate oxidase (ACO) proteins in Arabidopsis and rice using SWISS-MODEL [58]

In this study, the 3D protein structure of all SAM, ACS, and ACO genes and their ligand-binding site was predicted based on the homology model using the SWISS-MODEL database for predicting the protein-protein interactions (Figs. 3, 4, 5). The ligand-sites for S-adenosylmethionin and protein-ligand interaction profiler (PLIP) were observed in all Arabidopsis-SAM proteins and three rice-SAM proteins (*LOC_Os01g18860*, *LOC_Os01g22010,* and *LOC_Os0504510*) (Fig. 3). The ligand site of MES (2-(N-Morpholino)-ethanesulfonic acid) was observed in all predicted-ACS proteins except *AT1G01480* (Fig. 4). Also, the ligand-binding site of PLP (Pyridoxal-5- Phosphate) was found in the structure of ACO proteins. However, the binding sites of AAD ((2-Aminooxy-Ethyl)-[5-(6-Amino-Purin-9-YL)-3, 4-Dihydroxy-Tetrahydro-Furan-2-Ylmethyl]-Methyl-Sulfonium) and 2-Amino-4-(2-Amino-Ethoxy)-Butyric acid were observed only in Arabidopsis-ACS proteins (Fig. 5). For most ACO proteins, the ligand-binding site was not predicted; however, the ion-binding sites (Fe, zinc, and nickel ion) were observed in some ACO proteins (Fig. 5). According to the 3D structure and ligand type, *AT2G19590* was most similar to *LOC_Os09g27750* and *LOC_Os09g27820*, and also, *AT3G46500* was similar to *LOC_Os10g37899* (Fig. 5).

**Fig. 6:**
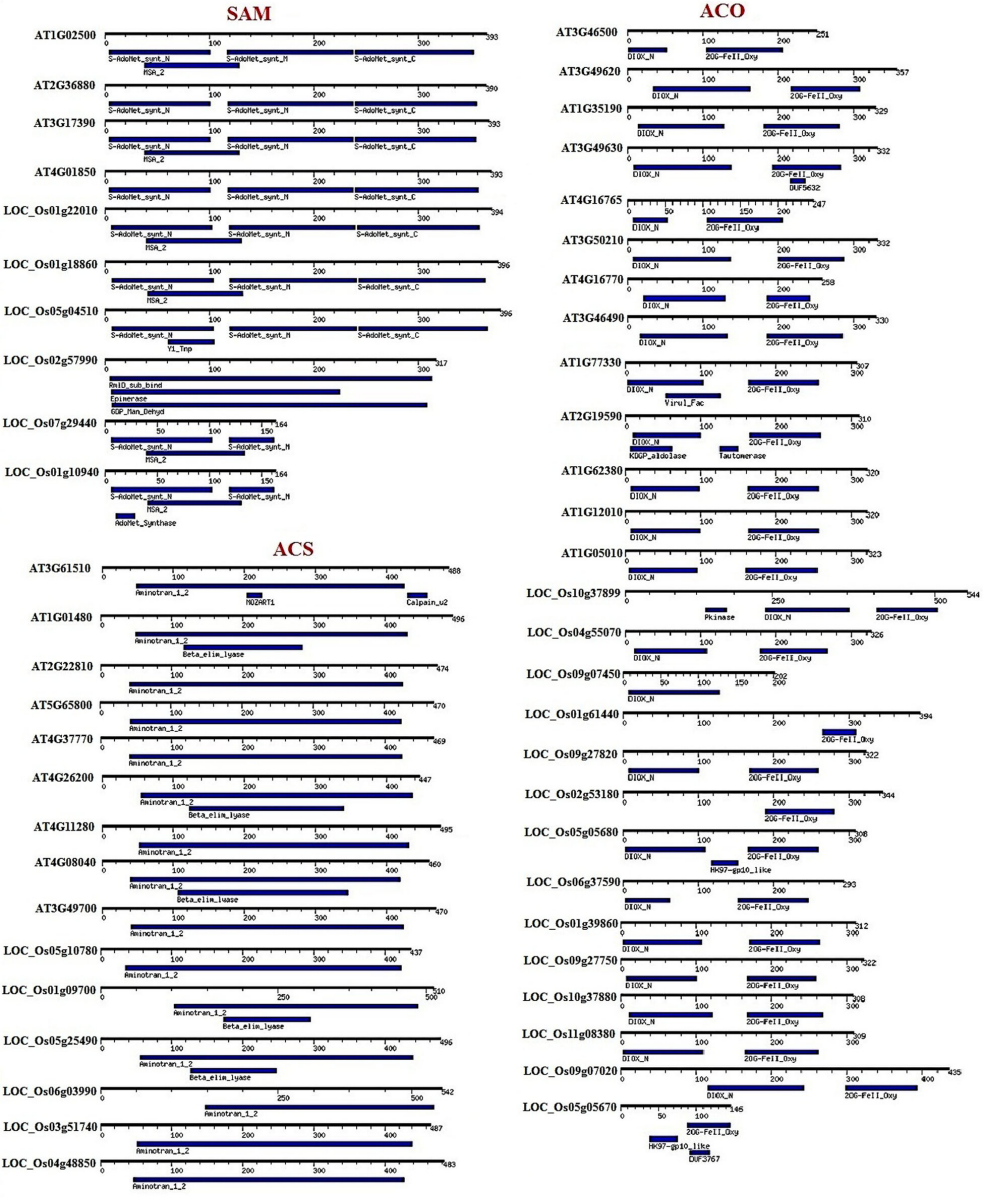
Motif and domain analysis of methionine adenosyltransferase (SAM), aminocyclopropane-1-carboxylate synthase (ACS), and aminocyclopropane-1-carboxylate oxidase (ACO) proteins in Arabidopsis and rice using the MOTIF Search program (https://www.genome.jp/tools/motif/)

The motif analysis for SAM, ACS, and ACO proteins was carried out using the MOTIF Search program (https://www.genome.jp/tools/motif/), separately (Fig. 6). According to the result of motif analysis, location and order of 3 motifs in SAM proteins were similar except for *LOC_Os02g57990*, *LOC_Os07g29440*, and *LOC_Os01g10940*. Moreover, motifs with different lengths and locations observed in *LOC_Os02g57990*, that the *LOC_Os2g57990* was predicted to have Golgi apparatus localization had more genetic distance than other rice-SAM genes (Table 1, Fig. 2, 6). Aminotron_1_2 motif was detected in all of the studied ACS proteins in Arabidopsis and rice, which are almost located in the same position. Besides, Beta_elim_lase was identified in *LOC_O05g25490*, *LOC_Os01g09700*, *At04g08040*, *At04g26200*, and *At01g01480* (Fig. 6). Two motifs were detected in most of the ACO proteins with identical order in Arabidopsis and rice; however, in some ACO proteins such as *At02g19590* and *LOC_Os05g05670*, proteins had two additional different motifs with various length and locations. The *At02g19590* and *LOC_Os05g05670* proteins with 310 aa and 157 aa length, respectively, were predicted as stable proteins and localized in the cytoplasm (Table 1, Fig. 6).

### Gene expression: Anatomy, development stages, biotic, and abiotic stresses and hormones treatment

**Fig. 7:**
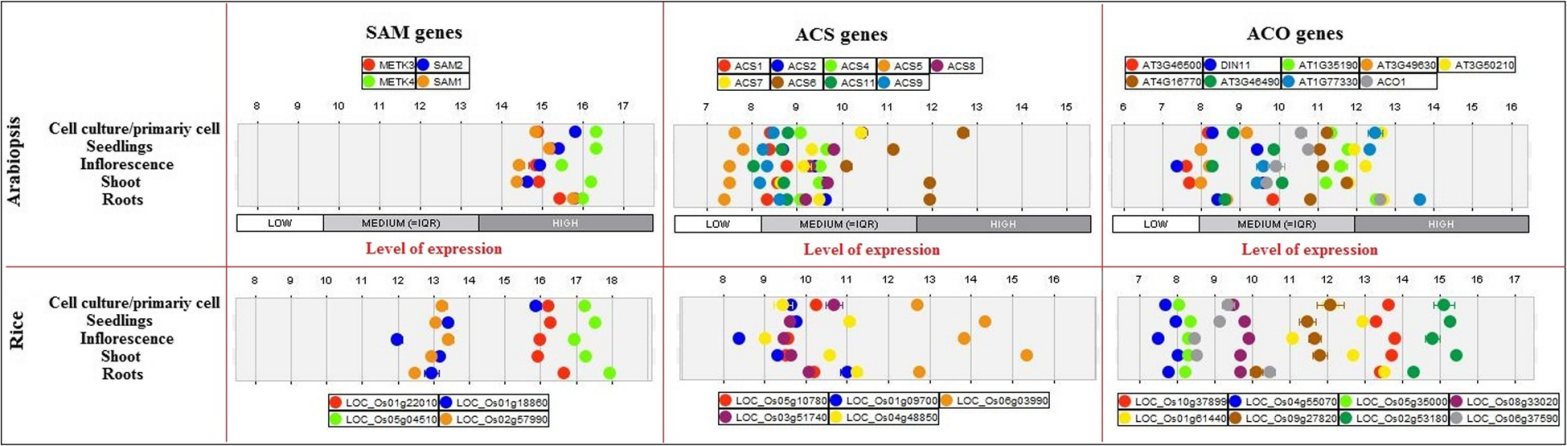
Expression level of ethylene biosynthesis genes in various tissues and organs of rice and Arabidopsis. The expression data were obtained from Affymetrix Arabidopsis ATH1 genome array using the Genevistigator database [59]

**Fig. 8:**
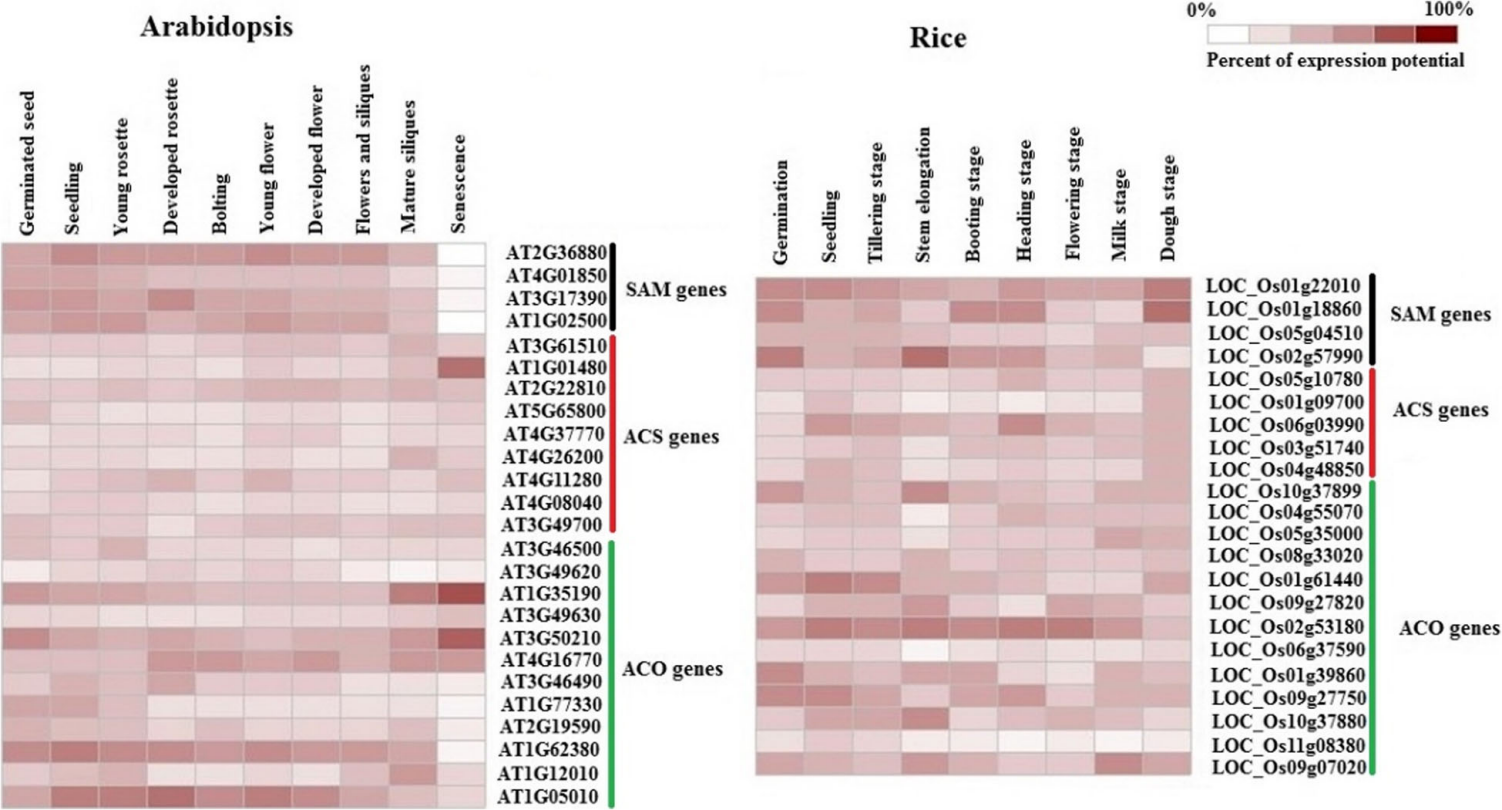
Expression patterns of ethylene biosynthesis genes at different development stages in rice and Arabidopsis. The expression data were obtained from Affymetrix Arabidopsis ATH1 genome array using the Genevistigator database [59]

**Fig. 9:**
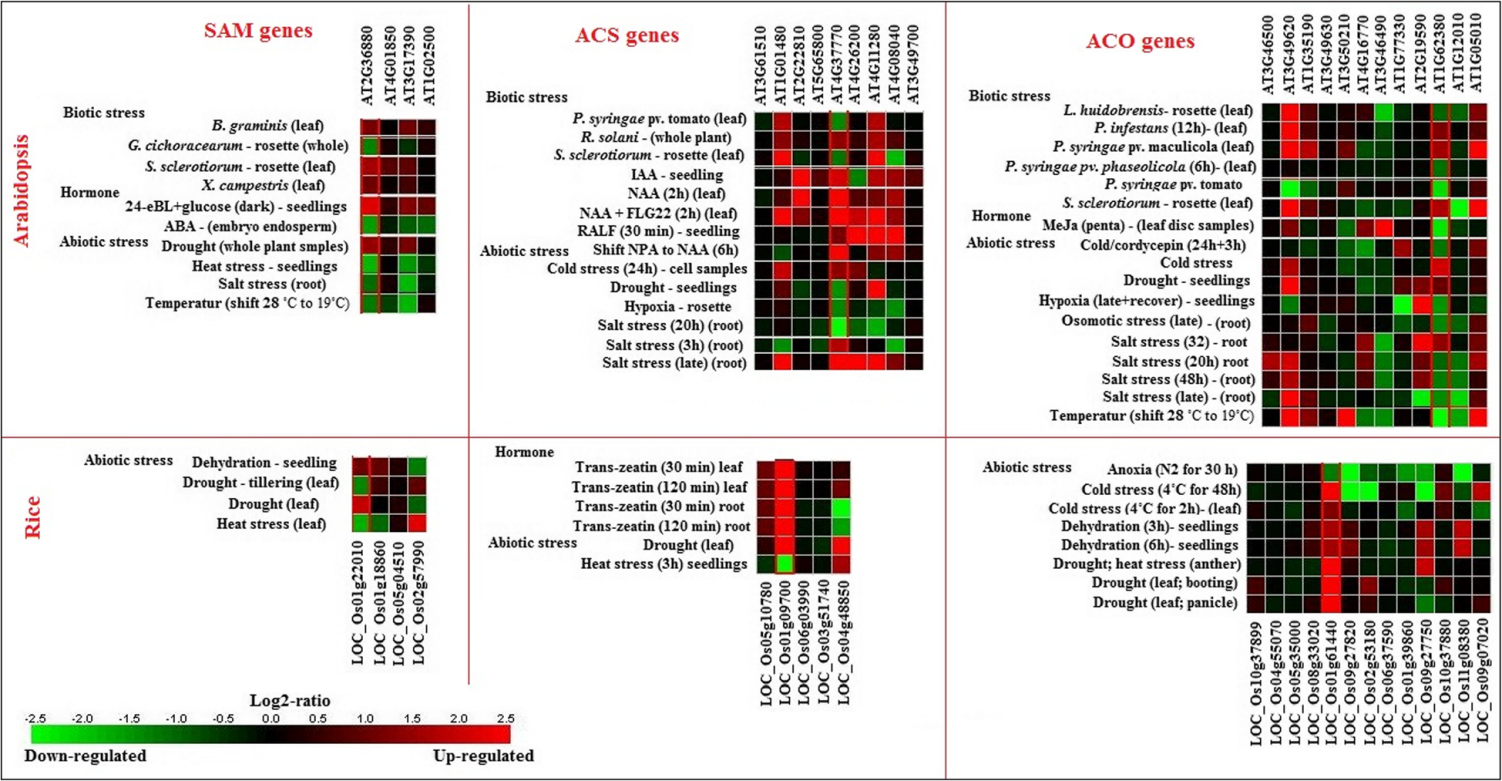
Differential expression of ethylene biosynthesis genes in rice and Arabidobsis under different biotic and abiotic stresses and hormones treatment. The expression data were obtained from Affymetrix Arabidopsis ATH1 genome array using the Genevistigator database [59]

The expression patterns of SAM, ACS, and ACO genes were evaluated in different tissues, organs, growth and development stages, as well as, under biotic and abiotic stresses and hormones treatment in Arabidopsis and rice via microarray data analysis available online using the Genevestigator database (Figs. 7, 8, 9). SAM genes including SAM1, SAM2, METK3, and METK4 high expressed in the primary cell, seedling, inflorescence, shoot and root in Arabidopsis, whereas, *LOC_Os01g22010* and *LOC_Os05g04510* showed a high level of expression in studied tissue and organs of rice, also, *LOC_Os01g18860* and *LOC_Os02g57990* expressed in medium level (Fig. 7). In Arabidopsis, *ACS1*, *ACS2*, *ACS4*, *ACS5*, *ACS8*, *ACS7*, *ACS6*, *ACS11,* and *ACS9* showed exclusive expressions in different tissues and organs. Most of the ACS genes showed medium expressions in studied tissue and organs except *ACS5* that expressed in low level in all studied tissues and organs, and *ACS6* showed a high level of expression in the primary cell, shoot, and root (Fig. 7). In rice, *LOC_Os06g03990* displayed a high level of expression in studied tissues and organs, whereas, *LOC_Os05g10780*, *LOC_Os03g51740*, *LOC_Os04g48850,* and *LOC_Os01g09700* showed a medium level of expression in all studied tissues and organs except *LOC_Os01g09700* had a low level of expression in the inflorescence (Fig. 7). Most of the ACO genes expressed in medium level, *DIN11* and *At03g46500* showed the lowest level of expression in inflorescence, as well as, *At3g46500* had a low expression level in shoot among the studied ACO genes in Arabidopsis. Besides, *At01g77330*, *ACO1*, *At03g50210*, and *At01g35190* expressed at a high level at the shoot, also *At01g77330* had a high level of expression in the primary cell and seedling. Moreover, *At03g50210* demonstrated a high level of expression in the primary cell and inflorescence (Fig. 7). Nine studied ACO showed various expression levels in rice, according to the obtained results *LOC_Os10g37899*, and *LOC_Os02g53180* revealed the highest level of expression, whereas, *LOC_O04g55070* and *LOC_O05g35000* showed low expression levels in studied tissue and organs of rice (Fig. 7). It could be concluded that almost all SAM, ACS, and ACO expressed in studied tissue and organs, but at different levels. The results of SAM, ACS, and ACO genes expression were investigated in different growth and development stages (Fig. 8). The Arabidopsis-SAM genes are mostly expressed in the germination stage, while two rice-SAM genes (*LOC_Os01g18860* and *LOC_Os01g22010*) are highly expressed in the ripening stage. Furthermore, Arabidopsis-*ACS2* more induced than other ACS genes that showed high expression in the seeds (Fig. 8). Among ACO genes, *At01g35190,* and *At03g50210* were more up-regulated than others and they had high expression in the seeds. Regarding the gene expression patterns, SAM genes were more involved in the rice-ripening stage, while in Arabidopsis, ACS and ACO genes were contributed in maturity (Fig. 8).

The expression of SAM, ACS, and ACO genes was studied under biotic and abiotic stresses and hormones treatment in Arabidopsis and rice through the existence of microarray data (Fig. 9). The *At02g36880* (SAM gene) showed high differential expression under stress conditions in Arabidopsis. The *At02g36880* gene is up-regulated in 24-CBL+ glucose (dark) in hormone treatment at the seedling stage, but it down-regulated under heat (seedling), salt (root), and temperature, as well as, *G. cichoracearum* (biotic stress). In rice, the *LOC_Os01g22010* gene up-regulated under drought (leaf) and dehydration, on the contrary, it down-regulated in the drought (tillering) and heat stresses, interestingly *LOC_Os02g57990* displayed vice-versa pattern in studied conditions (Fig. 9). Considering to filtration of ACS genes in Arabidopsis, *At04g37770* demonstrated various expressions under different stress conditions, where this gene is up-regulated under IAA (seedling), NAA, NAA + FLG22, RALF and shift NPA to NAA, while it down-regulated in some abiotic stresses. Besides, the gene expression profile of some ACS genes illustrated that *LOC_Os01g09700* gene is especially up-regulated at the different time courses of trans-zeatin treatment and drought stress condition, but it showed down-regulation under heat stress (Fig. 9). Regarding the expression pattern of ACO genes in Arabidopsis, the *AT03g49620* and *At01g62380* genes showed up-regulation and down-regulation in most of the studied conditions, respectively. While, *LOC_Os01g61440* up-regulated under abiotic stresses consisting of the cold, dehydration, drought, and heat condition in rice (Fig. 9).

It is worth noting that some of the studied genes with similar expression patterns under particular stress had a more close evolutionary relationship with each other and were categorized in the same groups. For instance, some ACS genes including *At04g37770*, *At04g26200,* and *At04g11280* genes are up-regulated under salt stress (late, root) which belonged to group I in phylogeny analysis, also *LOC_Os01g09700* and *LOC_Os04g48850* showed a high level of expression under drought conditions that categorized in group II (Figs. 2, 9). Moreover, among ACO genes *At01g62380* and *Ato1g12010* down-regulated under temperature (28 °C to 19 °C), and *LOC_ Os09g27820*, *LOC_ Os02g531810* and *LOC_ Os09g27750* down-regulated under cold stress (4 °C for 48 h) that clustered in group III regarded to phylogeny analysis (Figs. 2, 9). It seems that similar expression patterns of these genes in exclusive stresses were associated with the alike cis-elements underlying the promoter region of these genes. It reveals that the transcript of these genes adjusted with the identified transcription factors in the same conditions. Thus, gene expression study under various conditions showed environmental signals and stresses influence on the regulation of ethylene biosynthesis pathway, the achieved results could help to figure out how the underlying pathway gene networks were organized and adjusted in various tissues, organs, developmental stages, and stress conditions.

### Prediction the miRNA targets

In the present study, the sites of microRNAs (miRNA) were predicted using published miRNA sequences of psRNATarget server for Arabidopsis and rice (Table 2). The result of miRNA targeting the transcript sequences of SAM, ACS, and ACO genes revealed that *SAM1* (*AT1G02500*) from Arabidopsis was targeted by ath-miR843 while osa-miR1858 targeted two rice-SAMs (*LOC-Os01g22010* and *LOC-Os05g04510*) transcripts. Two rice-ACOs and one Arabidopsis-ACO contained the link-sites of ath-miR3933, osa-miR5809, and osa-miR531, respectively. All microRNAs inhibition involved the transcript cleavage. In our study, the complex between published miRNAs with transcript sequences of ethylene biosynthesis genes in *A. thaliana* and *O. sativa* were identified that would be helpful to understand the regulation the gene expression after the transcription process.

**Table 2:** Putative miRNAs targeted the transcripts of ethylene biosynthesis genes

miRNA ID	Target ID	miRNA sequence (3-5)	Target position	Expectation	Inhibition
ath-miR843	*AT1G02500* (*SAM1*)	AGGUUACUUCGAGCUGGAUUU	1333–1353	3	Cleavage
ath-miR843	*AT2G22810* (*ACS4*)	AGGUUACUUCGAGCUGGAUUU	675–694	2.5	Cleavage
ath-miR159a	*AT2G22810* (*ACS4*)	AUCUCGAGGGAAGUUAGGUUU	437–457	3	Cleavage
ath-miR159a	*AT4G37770* (*ACS8*)	UCCUCGAGGGAAGUUAGGUUU	458–478	1.5	Cleavage
ath-miR3933	*AT1G77330* (*ACO5*)	GGCUCAGCAGUAAAACGAAGA	739–759	2.5	Cleavage
osa-miR1858	*LOC_Os01g22010* (SAM)	CGGGGUGAGGCAGGAGGAGAG	567–587	3	Cleavage
osa-miR1858	*LOC_Os05g04510* (SAM)	CGGGGUGAGGCAGGAGGAGAG	570–590	3	Cleavage
osa-miR5809	*LOC_Os05g05680* (ACO)	CGACACCAGCGGCCGCUGCU	802–821	3	Cleavage
osa-miR531a	*LOC_Os11g08380* (ACO)	UACCGCCGUGCGUCGGGGCCGCUC	900–923	3	Cleavage
osa-miR531b	*LOC_Os11g08380* (ACO)	GCCGUGCGUCGGGGCCGCUC	904–923	3	Cleavage

### Cis-regulatory elements in promoter site

Gene expression is broadly adjusted in the transcription phase, where the interactions amongst cis-regulatory elements and transcription factors in the promoter region of the genes which perform a crucial role. In other words, the cis-regulatory elements (CREs) as non-coding DNA are mainly located in upstream of genes, which are determined via transcription factors that control the gene expression in various conditions. Analyses of the promoter region of the induced genes led to the discovering of cis-acting elements, also the ethylene-responsive element-binding protein (EREBP) family that interacts with ethylene response factors (ERFs) and DNA [3]. Transcription factors related to the ERF family have been demonstrated to be engaged in several developmental processes [64–66], abiotic [67, 68], and biotic [69] stress responses. The upstream of studied genes (promoter site) was screened to identify the key cis-elements that regulate the gene expression under different conditions (Table 3). The AAACAAA sequence named anaero1 consensus was observed in the most promoter sites of ethylene biosynthesis genes of *A. thaliana* and *O. sativa*. The anaero1 consensus is one of the motifs found in the promoters of anaerobic genes involved in the fermentative pathway [76]. The binding sites of common transcription factors such as MYB, WRKY, and ABRE that control target genes in abiotic and biotic stresses were generally distributed in promoter sites of ethylene biosynthesis genes of *A. thaliana*. SORLIP1AT, SURECOREATSULTR11, ABRELATERD1, MYBCOREATCYCB1, and LS7ATPR1, discovered in promoter site of all the ethylene biosynthesis genes of A. *thaliana* genes that these cis-motifs are engaged to response light-induced cotyledon and root genes [70], sulfur-responsive element [71], dehydration [72], cell cycle phase-independent activation and salicylic acid [73], respectively. Besides, the binding sites of some key cis-regulatory elements such as BIHD1OS, CGACGOSAMY3 and GARE2OSREP1 that involved in disease resistance [78], sugar starvation [79] and gibberellin-responsive element (GARE) [81], respectively, were commonly distributed in promoter sites of ethylene biosynthesis genes of O. *sativa*.

**Table 3:** List of key cis-regulatory elements of promoter site of ethylene biosynthesis genes

Identifier	Sequence	Cover %		Annotation
		Arabidopsis	Rice	
SORLIP1AT	GCCAC	100	–	Over-represented in light-induced cotyledon and root common genes and root-specific genes [70]
SURECOREATSULTR11	GAGAC	100	–	Sulfur-responsive element [71]
ABRELATERD1	ACGTG	100	–	Responsive to dehydration [72]
MYBCOREATCYCB1	AACGG	100	–	Involved in cell cycle phase-independent activation
WBBOXPCWRKY1	TTTGACY	100	–	W box; WRKY
LS7ATPR1	ACGTCATAGA	100	–	A positive salicylic acid-inducible element [73]
XYLAT	ACAAAGAA	96.2	–	Involved in secondary xylem development and wood formation [74]
CCA1ATLHCB1	AAMAATCT	92.3	–	Involved in the phytochrome regulation [75]
ANAERO1CONSENSUS	AAACAAA	96.2	96.2	Involved in the fermentative pathway [76]
SITEIOSPCNA	CCAGGTGG	–	100	Resemble G-box; May contribute in part to transcriptional activation [77]
BIHD1OS	TGTCA	–	100	Involved in disease resistance [78]
CGACGOSAMY3	CGACG	–	100	Involved in sugar starvation [79]
E2F1OSPCNA	GCGGGAAA	–	100	Involved in actively dividing cells and tissue [80]
GARE2OSREP1	TAACGTA	–	100	Gibberellin-responsive element (GARE) [81]

### Potential phosphorylation and glycosylation sites

**Fig. 10:**
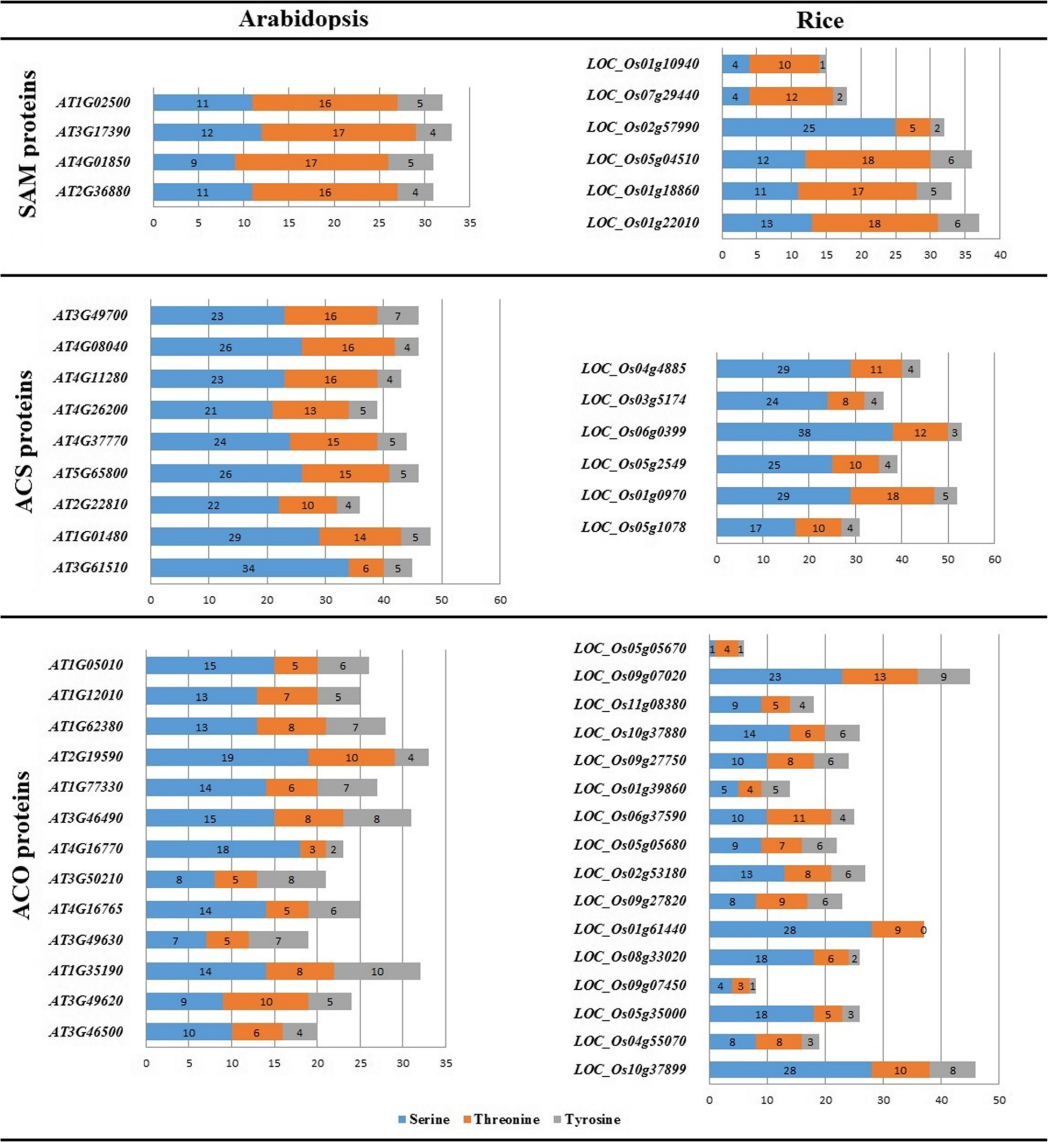
The predicted sites of phosphorylation in amino sequences of methionine adenosyltransferase (SAM), aminocyclopropane-1-carboxylate synthase (ACS), and aminocyclopropane-1-carboxylate oxidase (ACO) proteins in Arabidopsis and rice using NetPhos 3.1 server (http://www.cbs.dtu.dk/services/NetPhos/) [63]

Phosphorylation and glycosylation are the prevalent post-translational modification of proteins which could modify object site and activity of protein [82]. The potential phosphorylation sites of studied proteins were predicted based on the presence of serine, threonine, and tyrosine amino acids (Fig. 10). Phosphorylation is catalyzed by kinases that transmit a phosphoryl group commonly from ATP, but also from ADP to the hydroxyl group of particular Ser, Tyr, or Thr residues in their target proteins. Nevertheless, also His and both Asp and His in plant two-component signaling can be phosphorylated [83–86]. The result illustrated that the *LOC_Os05g05670* (as an ACO protein) had the minimum phosphorylation sites while the highest phosphorylation number (53 sites) predicted in *LOC_Os06g03990* (as an ACS protein). The predicted phosphorylation sites in SAM, ACS and ACO proteins of Arabidopsis ranged from 19 (*AT3G49630* as an ACO protein) to 48 (*AT1G01480* as an ACS protein). According to our findings, the ACO proteins were less phosphorylated than ACS and SAM proteins. This is likely that phosphorylation of ACS adjusts ethylene production was supported through the study that mutation of the C-terminal extension of ACS5 in Arabidopsis persuades the eto2-1 mutant to overproduce. The predicted-glycosylation sites within amino acid sequences of SAM, ACS, and ACO proteins were presented in table 4. All Arabidopsis-SAMs showed similar glycosylation patterns while the glycosylation patterns were very different in rice-SAMs and 50% of them were not predicted any glycosylation site. ACS proteins showed the highest glycosylation sites whereas *AT2G22810* (*ACS4*) had four predicted-glycosylation sites (as hyperglycosylated protein). The rice-ACO proteins showed the minimum predicted-glycosylation sites that 75% had no glycosylation site. Also, 38% of Arabidopsis-ACO proteins had no potential glycosylation site.

**Table 4:** The predicted N-glycosylation sites in amino sequences of methionine adenosyltransferase (SAM), aminocyclopropane-1-carboxylate synthase (ACS), and aminocyclopropane-1-carboxylate oxidase (ACO) proteins in Arabidopsis and rice using NetNGlyc 1.0 server (http://www.cbs.dtu.dk/services/NetNGlyc/) [62]

	*Arabidopsis thaliana*		*Oryza sativa*	
	Locus ID	Glycosylation site	Locus ID	Glycosylation site
SAM proteins	*AT2G36880*	2 (158, 233)	*LOC_Os01g22010*	2 (160, 235)
	*AT4G01850*	2 (158, 233)	*LOC_Os01g18860*	1 (236)
	*AT3G17390*	2 (158, 233)	*LOC_Os05g04510*	2 (161, 236)
	*AT1G02500*	2 (158, 233)	*LOC_Os02g57990*	0
			*LOC_Os07g29440*	0
			*LOC_Os01g10940*	0
ACS proteins	*AT3G61510*	1 (131)	*LOC_Os05g10780*	1 (189)
	*AT1G01480*	2 (132, 207)	*LOC_Os01g09700*	2 (264, 496)
	*AT2G22810*	4 (124, 199, 447, 456)	*LOC_Os05g25490*	1 (217)
	*AT5G65800*	2 (124, 199)	*LOC_Os06g03990*	2 (140, 282)
	*AT4G37770*	2 (124, 199)	*LOC_Os03g51740*	2 (46, 211)
	*AT4G26200*	2 (50, 214)	*LOC_Os04g48850*	2 (21, 204)
	*AT4G11280*	3 (27, 210, 229)		
	*AT4G08040*	3 (34, 122, 197)		
	*AT3G49700*	2 (124, 199)		
ACO proteins	*AT3G46500*	0	*LOC_Os10g37899*	0
	*AT3G49620*	1 (124)	*LOC_Os04g55070*	1 (307)
	*AT1G35190*	2 (3, 23)	*LOC_Os05g35000*	0
	*AT3G49630*	1 (99)	*LOC_Os09g07450*	1 (98)
	*AT4G16765*	0	*LOC_Os08g33020*	0
	*AT3G50210*	1 (98)	*LOC_Os01g61440*	0
	*AT4G16770*	1 (194)	*LOC_Os09g27820*	0
	*AT3G46490*	1 (3)	*LOC_Os02g53180*	0
	*AT1G77330*	0	*LOC_Os05g05680*	0
	*AT2G19590*	1 (110)	*LOC_Os06g37590*	0
	*AT1G62380*	0	*LOC_Os01g39860*	0
	*AT1G12010*	0	*LOC_Os09g27750*	0
	*AT1G05010*	1 (99)	*LOC_Os10g37880*	1 (178)
			*LOC_Os11g08380*	0
			*LOC_Os09g07020*	3 (65, 70, 205)
			*LOC_Os05g05670*	0

## Discussion

Ethylene is one of the simplest well-characterized plant hormones and ethylene biosynthesis including three simple steps, beginning from the amino acid methionine in both dicot and monocot plants [13]. Firstly, methionine is transformed to S-adenosyl methionine (SAM), which is afterward converted to 1-aminocyclopropane-1-carboxylic acid (ACC) via ACC synthases (ACS). Eventually, ACC is converted to ethylene through ACC oxidases (ACO) [87]. In this study, 26 and 28 engaging genes involving in the ethylene biosynthesis pathway, were predicted in *A. thaliana* and *O. sativa*, respectively. The selected SAM, ACS, and ACO genes in Arabidopsis and rice were variable in physicochemical properties including protein length, GRAVY value, aliphatic index, molecular weight, isoelectric points (pI), and instability index. The GRAVY values in involved-ethylene biosynthesis genes of Arabidopsis were more varied than rice. The GRAVY value associated with the solubility of proteins, and it could calculate the sum of hydropathy values [88, 89]. According to the predicted GRAVY value, ACS enzymes of Arabidopsis are more hydrophilic than ACS enzymes of rice. Besides, the ACOs of rice showed high variation based on physicochemical properties. Besides, the lowest and highest aliphatic indices observed in ACO and SAM rice predicted proteins, respectively. The aliphatic index is an important factor for the thermostability of proteins [90]. Research showed that Arabidopsis 14-3-3 protein exploits as positively adjusting the ethylene biosynthesis via increasing the ACS protein stability by the interaction with ACS proteins [55]. The proteins by high aliphatic index may have a greater half-life and they could be engaged in high reaction temperature [91].

Regarding the appearance and advancement of the genomic era, progressively, more genome sequences are released, which pave the way for evolutionary and comprehensive studies of any gene family from various species [82]. Our result based on studied predicted proteins indicated that involved-proteins in the ethylene biosynthesis pathway of rice had high variation than Arabidopsis. Lee and Yoon [20] indicated that the similarity of structure and the conserved regulatory motif discovered in both ACS proteins from these two plant species rice and Arabidopsis indicate the being of an evolutionally conserved mechanism, which underlies the ethylene biosynthesis regulation in rice and Arabidopsis. Also, the different ligand sites were observed in the predicted-3D structure of ACO and ACS proteins. Illuminating the biochemical and biological roles of proteins to determine their interacting partners, could be time-consuming and hardly implement by in vivo and/or in vitro approaches, besides most of the recently sequenced proteins will have unclear functions and structures as well. Although, computational approaches for predicting protein–ligand binding sites suggest an alternating practical solution. Therefore, it is momentous to discover these key sites to understand the protein function [92–94]. MES (2-(N-Morpholino)-ethanesulfonic acid) binding site was observed in all predicted-ACS proteins except *AT1G01480*, while the binding sites of AAD ((2-Aminooxy-Ethyl)-[5-(6-Amino-Purin-9-YL)-3,4-Dihydroxy-Tetrahydro-Furan-2-Ylmethyl]-Methyl-Sulfonium) and 2-Amino-4-(2-Amino-Ethoxy)-Butyric acid just observed in Arabidopsis-ACS proteins. The structure of the ligand-binding site can influence the protein function, protein evolution, and protein-protein interaction [95].

Ethylene plays a main role in the senescence and fruit ripening initiation, also boosts the transcription and translation of responsive genes engaged to fruit softening, cell-wall metabolism, and membrane metabolism, via switching on the ethylene signaling transduction [89, 96, 97]. The results of gene expression demonstrated that SAM, ACS, and ACO genes were differentially induced in plant development stages and they had different expression patterns in monocots and dicotyledonous in response to stresses. *ACS2* gene of Arabidopsis is more induced than other ACS genes showing high expression in seeds. In Arabidopsis, ACS transcripts have been illustrated in etiolated seedlings, roots, stems, leaves, siliques, and flowers [18, 98, 99]. Each of the multigene family is differentially expressed for the time of auxin treatment, wounding, and ripening [100]. For example, *LE-ACS4,* and *LE-ACS2* genes are expressed at the ripening time in tomato [101], persuaded in mature green fruits after treatment by exogenous ethylene [101, 102] and over induced upon pericarp tissues wounding [103]. Some ACS genes including *At04g37770*, *At04g26200*, and *At04g11280* genes were up-regulated under salt stress. Lelièvre et al. indicated that expression of the ACC synthase gene is controlled through ethylene only during/ after chilling treatment, but the expression of the ACC oxidase gene could be regulated separately through either ethylene or chilling [104]. As already noted, in Arabidopsis, various abiotic stresses often enhance ethylene biosynthesis by enhancing the transcription of distinct subsets of ACS genes. Transcript levels of the ACS6 gene elevate in response to ozone [105]. *ACS2*, *ACS9*, *ACS6*, and *ACS7* are induced during hypoxia [106], but the expression of all the ACS genes decreased under anaerobic conditions in Arabidopsis [98]. Nevertheless, the transcript levels of separate subsets of the ACS genes enhance in response to osmotic stress, drought, high temperatures conditions, and after wounding [98, 99]. Gene expression is broadly adjusted in the transcription phase, where the interactions amongst cis-regulatory elements and transcription factors in the promoter region of the genes which perform a crucial role. The binding sites of important transcription factors including ABRE, MYB, and WRKY that regulate target genes under stresses were generally distributed in promoter sites of ethylene biosynthesis genes of *A. thaliana*. Considering the regulatory role of these elements could distinguish much of plant stress response by these elements existence [46, 107, 108]. Also, different cis motifs including sulfur-responsive element, dehydration, and hormone (salicylic acid, gibberellin, and abscisic acid) responsive elements were observed in upstream of SAM, ACO, and ACS genes. Cis-acting elements are particular binding sites for proteins that engaged in the initiation and regulation of transcription, which is suppressing or activating the gene transcription in response to altering growth conditions and different environmental stress [109]. Our results indicated that the most SAM and ACO genes were down-regulated in response to abiotic stresses that various factors such as type of cis-regulatory elements may affect the expression patterns. Collectively, the current study revealed that involved genes in the ethylene biosynthesis pathway play key roles, not only in regulating development stages such as the ripening stage but also in regulating the response to abiotic and biotic stresses tolerance.

The result of miRNA targeting the transcript sequences of SAM, ACS, and ACO genes showed that ath-miR843 and osa-miR1858 play a key role to regulate the post-transcription modification of SAM genes in Arabidopsis and rice, respectively. The ath-miR843 involves in response to low-oxygen (hypoxia) stress [110], and osa-miR1858 is one of the mirRNA that is related to rice grains development [111]. Also, the target site of ath-miR159a was found in the transcript sequence of *AT2G22810* and *AT4G37770* as ACS genes. MIR159a is a key microRNA that targets mRNAs coding of MYB proteins that bind to the regulative site of floral meristem identity gene LEAFY [112], also ath-miR159a involved in hypoxia stress [110]. The prediction result of the post-translation modification showed that ACS proteins were more phosphorylated and glycosylated. Phosphorylation and glycosylation are the prevalent post-translational modification of proteins which could modify object site and activity of protein [82]. Phosphorylation, as one of the most plentiful post-translational modifications, plays the main role in plant metabolism and signal transduction via modifying protein interactions, protein activities, or subcellular location [24, 86, 113–115]. Regarding evidence, it seems that the biosynthesis of ethylene is adjusted by phosphorylation events that probably affect the ACS protein turnover. Working on the usage of phosphatase inhibitors and kinase in tomato tissues and suspension cell cultures demonstrated that phosphorylation influence the activity and/or turnover of ACS [116]. Thus, it seems that ACS phosphorylation preserves the protein from the destruction that in turn may lead ACS to accumulate and ACS activity to enhance, considering for the burst of ethylene production via ripening fruit [117], noteworthy, *LeACS2* protein of tomato has been discovered to be phosphorylated in response to wounding [117]. The glycosylation could make alterations to the stability of the protein [118] and protein’s molecular weight [119]. To sum up, it seems the ethylene biosynthesis proteins from Arabidopsis were more glycosylated than rice’s proteins. Some studies highlight the possibility of posttranslational regulation of ACS [115, 118].

## Conclusion

Nowadays, computational analysis plays a substantial role in plant science. Appropriate computational approaches coupled with suitable databases are fundamental for detecting, organizing, integrating data information content furnishing novel insights into the involved genes in important pathways and biological systems as well. Ethylene is a gaseous hormone that controls various physiological pathways. In this study, the involved genes in ethylene biosynthesis were evaluated using available bioinformatics tools in Arabidopsis and rice. Results revealed that involved-enzymes in ethylene biosynthesis had more variation based on physic-chemical characters and patterns of gene expression, protein structure, post-translation modification, and type of cis-regulatory elements. The genes in the ethylene biosynthesis pathway of rice had high variation than Arabidopsis indicated that probably SAM, ACS, and ACO genes of dicots such as Arabidopsis are derived from monocot such as rice. All SAM, ACS, and ACO genes are expressed in studied tissue and organs, but at different levels. SAM genes are more involved in the rice-ripening stage, while in Arabidopsis, ACS and ACO genes are contributed in maturity. Also, the SAM, ACS, and ACO genes expression of rice in different tissue and organs demonstrated more variation in comparison with the Arabidopsis genes. Regarding the post-translation modification result, the ACO proteins were less phosphorylated than ACS, and SAM proteins, and it seems the ethylene biosynthesis proteins from Arabidopsis were more glycosylated than rice’s proteins that can affect the protein activity, or subcellular location. Overall, the current study described that involved genes in the ethylene biosynthesis pathway play the key roles in controlling the response to abiotic and biotic stresses tolerance that various factors such as PPIs, type of cis-regulatory elements, and post-transcription/translation modifications could affect their expression. Our study was the first in silico and review study which widely assessed SAM, ACS, and ACO genes that are involved in ethylene biosynthesis and it provided an expanded landscape of computational analysis for further dissection and functional characterization of SAM, ACS, and ACO genes.

## Data Availability

The datasets and raw data are available from the corresponding author on reasonable request.

## Abbreviations

PGRs: Plant growth regulators miRNA Micro-RNA SAM S-adenosylmethionine ACC 1-Aminocyclopropane-1-carboxylate ACO ACC oxidase CDPK Calcium-dependent protein kinase MAPK Mitogen-activated protein kinase ERF Ethylene response factor TFs Transcription factors CAREs Cis-acting regulatory elements MW Molecular weight pI Isoelectric point GRAVY Grand average of hydropathy PPI Protein-protein interaction
